# Using Transcriptomics and Cell Morphology Data in Drug Discovery: The Long Road to Practice

**DOI:** 10.1021/acsmedchemlett.3c00015

**Published:** 2023-03-22

**Authors:** Lavinia-Lorena Pruteanu, Andreas Bender

**Affiliations:** † Department of Chemistry and Biology, North University Center at Baia Mare, Technical University of Cluj-Napoca, Victoriei 76, 430122 Baia Mare, Romania; ‡ Research Center for Functional Genomics, Biomedicine, and Translational Medicine, “Iuliu Haţieganu” University of Medicine and Pharmacy, 400337 Cluj-Napoca, Romania; § Centre for Molecular Informatics, Department of Chemistry, University of Cambridge, Lensfield Road, Cambridge CB2 1EW, United Kingdom

**Keywords:** drug discovery, transcriptomics, gene expression, cell painting, cell morphology, assay predictivity

## Abstract

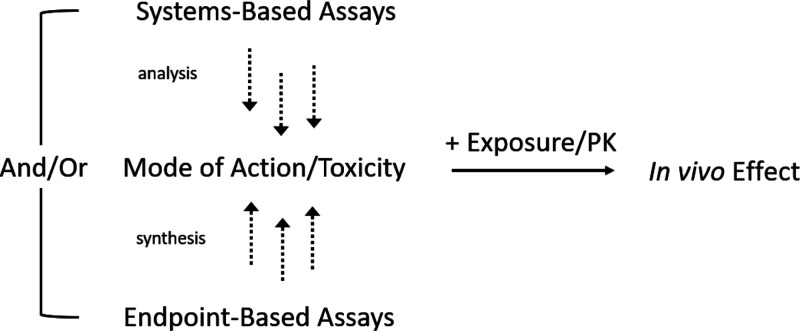

Gene expression and cell morphology data are high-dimensional biological readouts of much recent interest for drug discovery. They are able to describe biological systems in different states (e.g., healthy and diseased), as well as biological systems before and after compound treatment, and they are hence useful for matching both spaces (e.g., for drug repurposing) as well as for characterizing compounds with respect to efficacy and safety endpoints. This Microperspective describes recent advances in this direction with a focus on applied drug discovery and drug repurposing, as well as outlining what else is needed to advance further, with a particular focus on better understanding the applicability domain of readouts and their relevance for decision making, which is currently often still unclear.

Gene expression and cell morphology are high-dimensional biological readouts of much recent interest for drug discovery.^1−3^ One major reason for this is an unsatisfactory situation with respect to *in vivo*-relevant and translational early-stage assays, with clinical predictivity for efficacy and safety endpoints.^4−7^ At the same time, technological advancements in assay technologies, from Sanger sequencing to microarrays and more recently RNA-Seq and derived technologies such as DRUG-Seq,^8^ TempO-Seq,^9^ and others, have made data generation of gene expression data much more facile on a large scale. On the cell morphology side, assays such as Cell Painting^10^ have achieved a similar state of progress (e.g., related to relative assay standardization and data analysis methods) ca. 10–15 years later.

The potential (but to be proven) advantage of the above technologies from the practical side is their general applicability—given that biological readouts are generated on a “systems” scale (with this term being admittedly somewhat undefined), readouts obtained are potentially of interest for a wide variety of efficacy- and safety-related *in vivo* endpoints. However, such systems-wide readouts are generally obtained in a hypothesis-free (as opposed to hypothesis-driven) manner, hence describing general *variance* between systems instead of biological variables and endpoints of particular interest that have been defined from the onset. In practice, this means that the predictive validity^4,5^ of such wide endpoints is not defined *ab initio*, leading to the need to validate the predictive utility of a readout in a practically relevant drug discovery setting.

This Microperspective will give examples of the above readout domains, gene expression and cell morphology, and their recent applications in the drug discovery context. The following text will be structured by readout type, describing first gene expression readouts and then cell morphology readouts. Within each section, there will be first a brief discussion about technology developments of each readout type, followed by sections on efficacy and repurposing, safety, and mode of action analysis of compounds.^11^

Some general requirements for omics data to be used for efficacy- and safety-relevant decision making are summarized in a generic way in Table 1, with details depending on each particular case in practice.

**Table 1:** Requirements for Biological Endpoint Data to Make Practical Impact in Drug Discovery Projects

**Endpoint property**	**Minimum threshold**	**Best-case scenario**
Reproducibility	Intergroup variance is larger than intragroup variance	Consistently reproducible readout
Predictivity of signal to *in vivo* endpoints	Predictivity for subgroup of population	Predictivity across whole population (between and within subtypes)
Causality	Correlation-based marker, no causality	Causality present
Wide applicability/necessity of tailoring	Necessity of tailoring of assay/data processing setup for different *in vivo* endpoints	No tailoring needed for different *in vivo* endpoints
Standardized data processing pipeline	Data processing pipeline sufficiently standardized for decision making in some areas	Data processing pipeline standardized across all endpoints
Interpretability/allows inclusion of prior information	No inclusion of prior information possible	Inclusion of all desired prior information possible (e.g., pathway information)
Speed, cost	Practically feasible cost-wise for some projects, in plausible timeframes	Competitive in cost and time domain across all projects

## Transcriptomics Data

### Technology

In more recent history of gene expression profiling, microarrays established themselves in the early 2000s as a sufficiently low-cost and high-enough-throughput tool,^12,13^ which has resulted in viable commercial offerings such as the Affymetrix gene expression chips in particular. However, for profiling large numbers of compounds, costs were still prohibitive, which led to several advances, such as the L1000 platform,^14^ which provided again a decrease in cost by about 2 orders of magnitude, although it only provided information on the expression of around 1000 predefined genes. A scientifically separate route taken somewhat later in time involved targeted technologies often based on RNA-Seq, which usually involve simplified sample preparation (not requiring RNA isolation) and multiplexing, and which were generally amenable to automation. These technologies include RASL-Seq,^15^ and more recently also TempO-Seq^16^ and DRUG-Seq.^8^ Different recent methods have different trade-offs. For example, PLATE-seq^17^ allows samples in 96-well plates to be profiled in high throughput; however, it requires lengthy RNA purification steps, and the 96-well format is not sufficiently high-throughput for many practical situations. Currently with techniques such as DRUG-Seq, profiling costs per sample (including sample preparation and sequencing) on the order of dollars can be achieved, assuming the setup is in place, allowing also high-throughput automation.^8^ Developments in technologies in some cases went hand-in-hand with the availability of public data, such as in the form of the Connectivity Map^18^ and L1000 data sets.^14^ However, for RNA-Seq data, until the current time, no such data set is available in the public domain.

New methods, of course, need to be validated, both in the absolute sense (whether the readout obtained is of use for the intended decision to be made in a drug discovery project) as well as in a relative sense (whether a new technology agrees with previous technologies), and given the pace with which technology develops, this is in practice often a difficult aim. This is an immense pain point in the pharmaceutical context, both in the fundamental sense (“Which readout now gives me a predictive signal?” ^6,7^) as well as the practical sense (in order to establish databases and workflows in a consistent manner, without the requirement to establish processes, and comparisons to existing data, every few years).

Many of the above technologies have hence been subject to extensive validation studies. The publication establishing DRUG-Seq^8^ concluded, based on a data set of 433 compounds, that transcription profiles successfully grouped compounds into mechanism-of-action clusters based on their intended targets, although in this analysis, mechanism-of-action was not defined on a selective on-target basis (which is also often too simplified a view of compound action) but rather a broader modulation of biological function category. A subsequent study on the same technology^19^ used an open-source analysis pipeline which also shows “high reproducibility and ability to resolve the mechanism(s) of action for a diverse set of compounds”, and which was applied to NMDA receptor modulators. It would have been interesting to show in that study whether clinically efficacious NMDA modulators (such as ketamine) could be distinguished from those that turn out to be not efficacious in the clinic, which would be the holy grail of such readouts.

Some comparisons have been used in the context of safety assessment, such as for the New Approach Methodologies (NAMs; i.e., non-animal)-based hazard characterization of environmental chemicals using TempO-Seq in an MCF7 cell model.^20^ The authors conclude that data was reproducible, that aggregating genes into gene signatures is beneficial, and that this was the case in particular for concentration–response analyses, where previous reference data could be reproduced (i.e., the pathway gene expression signal corresponded to the most sensitive on-target assay activity from previous profiling). Including concentration is probably where safety is currently more advanced than when it comes to efficacy. TempO-Seq was validated in another study,^16^ which used 45 purified RNA samples from the livers of rats exposed to chemicals that fall into one of five modes of action, where it was found that microarrays and TempO-Seq capture the most variability in terms of mode of action, while here RNA-Seq had higher noise and larger differences between samples within a mode of action (which, however, is not bad, given that there is an individual aspect to compound pharmacology, beyond any annotated, labeled mode of action).

From the practical data analysis side, taking batch effects into account is crucial, since due to both biological variation and slight variation in assay protocol results may differ. Different methods have been compared recently,^21^ finding that the limma method correcting for two principal components showed the best performance.

More recent technologies include single-cell as well as spatially and time-resolved transcriptomics.^22−25^ Those technologies have led to pipelines for, e.g., drug repurposing based on single-cell data,^26^ a better understanding of the tumor microenvironment in lung cancer,^27^ and insights into early development of human embryos^28^ as well as other organisms. However, in addition to high cost and low throughput, there is often no clear path to practical deployment of many of those readouts in a decision-making context, since data generation, processing, and decision making have not yet converged on standardized pipelines to this end.

### Efficacy/Repurposing

Gene expression data has been used for a number of years, on a large scale since the advent of Connectivity Map, for indication discovery and repurposing, including compounds tested in animal models.^29^ While it is difficult to pinpoint where exactly gene expression data generally (or Connectivity Map data specifically) has been used for drug discovery, those cases do exist—for example, in particular subtypes of acute lymphoblastic leukemia (ALL), gene expression data suggested HDAC inhibitors as a treatment,^30^ which were later found to be efficacious in *in vivo* mouse models.^31^ Given that many factors influence decisions in a drug discovery project, it is difficult to identify gene expression data as *the* true starting point for a project—but ample evidence exists that it is certainly *one* useful starting point.

More recent publications on the topic^32^ integrate human gene expression, drug perturbation, and clinical data, and in this case for hyperlipidemia and hypertension, which based on an analysis of >21,000 compounds was able to replicate 10 approved drugs and identify 25 drugs approved for other indications with effects on clinically relevant biomarkers, which hence provides a first indication for efficacy.

Methodologically, e.g., Chemical-Induced Gene Expression Ranking (CIGER)^33^ has been a recent development, which has been validated in the pancreatic cancer context, albeit only on cell lines, and comparison to other methods has not been performed in this study. Utilizing a successor of the original Connectivity Map, the Library of Integrated Network-based Cellular Signatures (LINCS), as a database, and with the aim to provide better benchmarking, recent work^34^ identified homoharringtonine as a potential treatment for liver cancer, which was validated *in vivo* using xenograft and carbon tetrachloride-induced liver fibrosis models. Translation to humans remains an open question, but the aim to establish better benchmarking approaches is laudable.

One of the companies that explored using transcriptomics data for in-house discovery early on is Janssen, which recently used L1000 data for 31,000 compounds^35^ from Janssen’s primary screening deck to assess the potential of transcriptomics data to identify similar compounds and to generate target activity predictions and the scaffold-hopping potential of the resulting hits. For several targets, high-performing predictive models for on-target activity were obtained (balanced accuracy values ≥80%), which were also prospectively validated for novel scaffolds. One of the key questions is for *which* targets suitable predictive signals can be found in gene expression data, which is not yet entirely clear generally across endpoints, also due to differences in data generation and processing.

One of the key potential advantages of using gene expression data for compound characterization is that the signal is intrinsically biologically meaningful in that genes have a meaning with respect to their functions in pathways and in the cell as a whole. That this can be exploited practically has been shown when selecting agents for differentiation therapy in leukemia,^36^ where compounds were able to de-differentiate leukemia cells to granulocytes, which has also been the mechanistic selection criterion for compounds. Likewise, the application of using transcriptomics data to select small molecules that influence cellular differentiation has advantages, in that it enables therapy to move from transcription factors (with usually unsuitable pharmacokinetics (PK) profiles) to small molecules. That this is feasible in practice has been shown in a study^37^ which proposed and successfully validated several small molecules for differentiating stem cells to cardiomyocytes, which were profiled on the genetic and proteomics levels.

#### Compound Combinations

Gene expression data can also be used for combination drug discovery , with the caveat that combination treatment transcriptomics data is usually not available, and that hence some synergy (or other combination) hypothesis is needed to select suitable combinations. In practice, combination treatment is often different from just the “sum” of the monotreatments when it comes to gene expression responses,^38^ which has also been exemplified, e.g., in the area of adaptogens in more detail.^39^ This makes the selection of compound combinations with the desired effect based on single-compound data not trivial; on the other hand, having *some* combination hypothesis, such as that activity on the same pathway (or complementary pathways), is still a starting point for experimental study.

On the cellular level, gene expression data for drug combination discovery has been used, e.g., in pancreatic cancer drug discovery,^40^ which was then validated prospectively by testing 30 compounds (and their combinations) on PANC-1 cells. Compounds suggested as combination agents with the standard therapy gemcitabine, based on the best-performing scoring system, showed on average 2.82–5.18 times higher synergies compared to compounds that were predicted to be active as single agents. Gene expression data has also been used successfully for explaining synergistic effects, such as for the combination of fucoxanthin and the phosphatidylinositol 3-kinase (PI3K) inhibitor LY-294002,^41^ where further mechanistic insight into compound action could be gained.

#### Prediction of Gene Expression

Gene expression data is not available for all compounds, and hence understandably recent approaches also aimed to predict transcriptomics changes based on chemical structure. This is a nontrivial exercise, given the high-dimensional output space and the relatively small number of data points available, which in addition possesses strong analogue bias.

One recent approach, termed MultiDCP,^42^ aimed to take differences between cell lines as well as dose into account when predicting gene expression changes as well as changes in cell viability upon compound treatment. The transformer-based method has shown, according to the authors, that predicted drug-induced gene expressions demonstrate a stronger predictive power than experimentally derived data itself for downstream tasks.

Generative models have become tremendously popular recently, and hence also generative models for generating compounds with a desired transcriptomics effect have been proposed.^43^ The method was validated by the similarity of generated molecules to known bioactives, which led to generally plausible results, and conditioning of the Generative Adversarial Network (GAN) employed on transcriptomics data was hence successful.

### Safety

While the above applications of using gene expression data related to indication discovery and repurposing are plentiful, given the inherent multidimensionality of safety endpoints and the relative lack of availability of data, maybe applications in this field stand to gain even more when it comes to identifying the most suitable compounds to progress to the clinic. The experience of the authors, with colleagues, based on gene expression readouts^44,45^ has led to the conclusion that “things are not easy”—due to the multidimensional nature of transcriptomics readouts (and the unclear link to decisions), noise in the data, and the different nature of every project with many unknowns.

Using omics data for safety is driven by both technology advances and also the insight that broad profiling, across chemical and target space, is a huge endeavor, which still will be limited severely across both domains, and where omics data is possibly able to generate a broader view, at least on the biological response side. Hence also agencies changed their approach in the past years, such as the U.S. Environmental Protection Agency (EPA), moving toward the generation and working toward the regulatory acceptance of omics data in risk assessment.^46^ Challenges to be tackled are of technological, scientific, data analysis, standardization, and interpretation nature, and beyond, before methods will be able to advance public health and regulatory decisions (requirements for which have been discussed in ref (47)).

Given that animal testing has not been allowed for consumer products in Europe for a number of years, animal-free NAMs have been explored heavily by related companies; however, it is largely not clear which assays to utilize for decision-making related to which endpoint in practice. A recent study^48^ provides one of the few “complete” approaches in this area, for a hypothetical product containing 0.1% coumarin in face cream and body lotion and excluding existing animal and human data on coumarin in the process. Plasma *C*_max_ was estimated using a physiologically based kinetic model for dermally applied coumarin, while systemic toxicity was assessed using a battery of *in vitro* NAMs to identify points of departure (PoDs) for a variety of biological effects and combined with ToxCast data, an *in vitro* cell stress panel, and high-throughput transcriptomics. The predicted *C*_max_ values for face cream and body lotion were lower than all PoDs, with a margin of safety higher than 100; thus genotoxicity was ruled out and both receptor panel and immunomodulatory effects at consumer-relevant exposures were negative, hence showing a first successful comprehensive case study in the area. Applicability to future compounds and applications contexts remains key to proceed further.

#### Point of Departure Modeling

“Points of Departure” (PoDs) are a concept from safety assessment, describing the concentration when a biological response (e.g., transcriptional changes) is observed, and some recent studies aimed to refine the state of the art in this area further. Basili et al.^49^ employed a method combining prior information with available data, using the Pathway-Level Information ExtractoR (PLIER) algorithm to identify latent variables (LVs) describing biological activity, and the authors then analyzed those LVs using the ToxCast pipeline. For 44 chemicals in MCF-7 cells, they showed that the workflow was able to discriminate between estrogenic and anti-estrogenic compounds. Approaches which combine data with prior knowledge will likely be key in the future, given the size of chemical and biological space and our inability to sample it properly.

A study in the ecotoxicology context^50^ comprising a total of 10 different compound interventions found that transcriptomics-based PoDs were mostly *lower* than apical PoDs; however, for extrapolations to some organisms this was not the case. One key finding was that the number of genes included in a PoD signature was significantly related to its robustness; in this particular study design, fewer than 15 differentially expressed genes were found likely to be unreliable for screening.

#### Time Series Analysis

While the above studies on PoD put transcriptomics changes in relation to concentration, for toxic endpoints also changes over time matter—in particular, genes can change transcription as a *consequence* of damage, or as a *causal factor*, which makes an important practical difference, such as in the construction of Adverse Outcome Pathways (AOPs). To investigate this in the context of non-alcoholic fatty liver disease (NAFLD), recent work^51^ used data for 28 steatotic chemicals with gene expression data measured at three time points and three doses to describe compound effect, and hence mechanisms leading to NAFLD, as a function of time.

Also in the drug-induced liver injury (DILI) area, a time-series analysis of transcriptomics data considering the Bradford–Hill criteria has been performed,^52^ which includes whether events are consistently observed in a certain temporal order, and hence this work introduces the concept of “first activation” as a data-driven means to generate hypotheses on potentially causal mechanisms. Data from TG-GATEs comprised time points from 3 h to 4 weeks post-treatment, and both known and potential novel mechanisms involving DILI were identified. In addition, transcription factor analysis was performed, also including prior information into the analysis of data. As with similar analyses of this type, data remains a challenge—whether inter-individual variation, the high dimensionality of readouts, lack of data across time points, or dependence of any results on the choice of specific parameters.

### Mode of Action Analysis

“Mode of action” is a term easily said, but it can conceptually mean many things (direct targets, downstream modulation, etc.), and on the other hand *really* understanding the mode of action of a compound (with all its polypharmacology etc.) in a given disease context is a severe challenge. Accordingly, also labeling data with “modes of action” is tricky, as further outlined in a recent review.^11^

The analysis of modes of action has been done with the original CMap publication,^18^ e.g., for Trichostatin A, which also has been revisited by other technologies, such as TempO-Seq.^9^ However, in the experience of the authors, there is still a lack of understanding where each readout contains a suitable signal, given a way to generate data and a particular data analysis pipeline, leading to a biased representation of case studies—and there is likely a reason that, e.g., tubulin inhibitors, or said Trichostatin A, feature so often in such case studies, namely that this is a signal that is more easily observable than others. The question remains which on-target and pathway activities are observable in a given readout type in practice—and if we are unable to describe that, then we are unable to understand whether activity has not been observed because the compound does not cause it or whether it *fundamentally cannot be captured by a certain type of readout*.

In order to combine transcriptomics *data* with existing *prior information*, recent work^53^ employed causal reasoning to benchmark existing algorithms (SigNet, CausalR, CausalR ScanR, and CARNIVAL), networks (OmniPath vs MetaBase) for a data set of 269 mode-of-action-annotated compounds. It was found from an ANOVA analysis that the combination of algorithm and network most significantly dictated the performance of causal reasoning algorithms, with the effect of the data source being less strong. All causal reasoning algorithms also outperformed pathway recovery based on input Differently Expressed Genes (DEGs), with performance being somewhat correlated with connectivity and biological role of the targets.

## Cell Painting Data

### Technology

Compared to gene expression profiling, cell morphology profiling grew to maturity for practical purposes probably about a decade later (say, the early 2010s, compared to the early 2000s, although those numbers depend heavily on use case and technology). One significant practical development was the *standardization* of cell morphology readouts in the form of the Cell Painting^10^ assay at the Broad Institute, which aimed to enable large-scale data generation, and which is hence as essential as, say, the development of a microarray provider on the transcriptomics level. An obvious difference is the establishment of one readout type (microarrays) in the form of a standardized *physical tool* provided by commercial vendors, and the other readout type (Cell Painting) in the form of a *publication*, with no *single* provider for such services available. The Cell Painting assay has been shown to be robust also with slight variations of protocol and reagent vendors being used,^54^ which in addition to low running cost per data point (once the experimental environment has been established) adds to its advantages. In analogy to transcriptomics data, public data sets have been generated on a large scale, which has recently been made available by the Broad Institute (https://jump-cellpainting.broadinstitute.org/).

Given its relative youth, the exploration of where cell morphology, and in particular Cell Painting data, can be used for drug discovery is still not very advanced. Some publications exist which determine best practices for data analysis from Cell Painting screens (reviewed recently^55^). One such study^56^ explored curve fitting at several levels of data aggregation and on computed metrics, and hit identification strategies based on single-concentration analysis included measurement of total effect magnitude and correlation of profiles among biological replicates. Overall, most of the methods achieved a 100% hit rate for the reference chemical and high concordance for 82% of test chemicals, indicating that hit calls obtained for this data set are robust across different analysis approaches. As with gene expression data, batch correction needs to be performed also with Cell Painting readouts. Given that Cell Painting is about 15 years more recent compared to microarray technologies, agreement on which methods to use is still in its infancy; a data set to this end has now been made public.^57^ A recent review^58^ summarizes the current state of the art when it comes to data analysis, with the authors describing it as moving from unsupervised and often clustering-based methods currently toward incorporation of more sophisticated machine-learning algorithms in the future. Still, there is some way to go when it comes to establishing best practices from data generation to analysis and decision-making.

### Efficacy/Repurposing

While gene expression data has been used in a large number of repurposing studies so far, Cell Painting data has generally not reached this stage yet. However, this will likely change in the future, given that data for a large number of existing drugs is now also in the public domain,^59^ so that corresponding workflows will likely be set up and experimentally validated.

Given that cellular systems can be modulated by compounds, but also via, e.g., overexpression of genes (as well as in other ways), of course data from multiple dimensions can be used for, e.g., virtual screening. One recent study^60^ employed cell morphology images from gene overexpression to identify matching images from the small-molecule side, thereby identifying modulators of three target genes which were experimentally validated. While the imaging data obtained can hence be reused for novel targets, it also needs to be said that only for a minority of genes in the currently available data could compound matches be found, and that also not in all cases were gene overexpression and compound modulation phenotypes matched, and that hence a positive selection of cases that successfully worked has been performed.

Cell Painting data has also been used for compound selection against dihydroorotate dehydrogenase (DHODH)^61^ as well as tubulin,^62^ where in particular in the latter case it is understandable that a suitable, visually apparent cellular phenotype for such compound selection exists.

For esophageal adenocarcinoma, Cell Painting data has been used for compound selection, leading to 51 validated and selective hits out of a total library size of 19,555 compounds.^63^ For the most potent and selective hits, namely elesclomol, disulfiram, and ammonium pyrrolidinedithiocarbamate, mode-of-action elucidation led to copper-dependent cell death, which was then proposed as a novel way of targeting esphageal adenocarcinoma in the future.

As in the case of transcriptomics, also generative models have been applied to cell morphology data.^64^ Models were conditioned on cell morphology profiles of 30,000 compounds using their Cell Painting morphological profiles as conditioning. The model generated plausible chemistry, which in some cases resembled compounds with the desired bioactivities in the ExCAPE database. However, truly prospective testing of the model with novel compounds has not been performed.

### Safety

As described for transcriptomics data, also cell morphology, and Cell Painting data in particular, has been under intensive evaluation for safety endpoints, such as by the EPA.^46^

The predictive value of a readout, given a particular assay setup and data processing pipeline, needs to be validated for every endpoint individually. This has been done for mitochondrial toxicity,^65^ using Cell Painting, gene expression, and compound structural information, for 382 chemical perturbants tested in the Tox21 mitochondrial membrane depolarization assay. Mitochondrial toxicants were found to differ from nontoxic compounds in morphological space, and when included in predictive models this combination of features improved model performance on an external test set of 244 significantly, thereby improving extrapolation to new chemical space.

For new modalities, such as PROteolysis TArgeting Chimeras (PROTACs), it is not clear how to profile compounds for safety, and the Cell Painting assay has been evaluated for this purpose recently as well.^66^ It was found that the signal contained in Cell Painting readouts with respect to mitochondrial toxicity of PROTACs was concentration-dependent, with 10 μM and 1 μM offering better signals for model generation than a concentration of 0.1 μM, and with the model based on 1 μM concentration data offering virtually perfect classification on a prospective test set.

On a wider scale, a recent study used Cell Painting readouts to predict 70 cell health phenotypes across a wide range of biology,^67^ and it was generally judged to be successful. The practical impact is that potentially endpoints are predictive of safety in the clinic, although this needs to be evaluated further.

Cell Painting has also been employed^68^ to assess combination effects of cetyltrimethylammonium bromide, bisphenol A, and dibutyltin dilaurate on four human cell lines, and it was found that bisphenol A exacerbates morphological effects of the other two compounds. Importantly, in this work effects have been found to be cell line-dependent, hence requiring the assay setup to be chosen carefully to be relevant for the *in vivo* situation one aims to predict.

### Mode-of-Action Analysis

Attempts to use cell morphology data to understand a compound’s mode of action date back a number of years, and early attempts included an integrated view of morphology-based readouts with computational target prediction, showing the complementarity of both readout types.^69^

Cell Painting data has been used in multiple studies so far for mode-of-action analysis, such as in recent work^70^ which found for a set of 10 well-represented MoA classes that the macro-averaged F1 score of 0.58 when training on only the structural data but increased to 0.81 when training on only the image data and 0.92 when training on both input data types together. Note that not all bioactivity classes might be as well-populated or as easily annotated with a “mode of action”, and hence also here some kind of self-selection has been performed in this study. The general finding, of improved classification performance, is nonetheless consistent with a study cited above,^65^ which in particular for novel scaffolds underlined the value of including Cell Painting readouts in predicting biological endpoints.

One of the earlier studies utilizing Cell Painting data for target prediction/virtual screening^71^ for glucocorticoid receptor translocation was able to predict assay-specific biological activity in two ongoing drug discovery projects based on *predefined* image features. Here, out of 535 assays, 5.8% and 8.0% could be predicted with AUC-ROC at 0.9 or larger using Macau and Deep Neural Networks as classification methods, while for an AUC-ROC value of 0.7 this number was 40.7% and 45.8%, respectively. More recent work^72^ has been able to utilize convolutional neural networks (CNNs) *on the images directly*, and this study (albeit on a different data set) was able to improve upon the above numbers, now predicting 32% of the 209 biological assays at high predictive performance (AUC > 0.9). This means that Cell Painting data has the potential of being predictive for those targets also in a prospective discovery situation and, if this proves to be true in the future, to potentially replace such assays which are currently run on individual targets.

Also combining information from multiple domains, such as chemical structure and imaging information, has been performed using Bayesian matrix factorization (BMF Macau) methods and compared to Random Forest.^66^ It was found that both methods performed similarly when ECFP fingerprints were used as compound descriptors. However, BMF Macau outperformed Random Forest in 69.20% of cases when image data was used as compound descriptors. As demonstrated also in some of the above studies, here cell morphology information added considerable value for predictions of relatively diverse chemistry, where cell morphology endpoints were similar despite different chemistry, such as when targeting β-catenin.^73^

To conclude, a wide variety of methods to generate omics data (here focused on gene expression and Cell Painting data) exists, but there is no general consensus (a) for which chemical structure and (b) for which biological type of data (generated using which particular assay setup) and (c) using which analysis method is relevant for decision making for (d) which type of clinically relevant endpoint. This is the subject of much ongoing research, some of which has been summarized above, and this also makes the concept of an “Applicability Domain” multidimensional (extending significantly beyond the chemical domain, where it is conventionally applied), and hence much more complex than in a single domain alone (since applicability in one domain is *conditional* on the setup across all other domains).

So where do transcriptomics and Cell Painting data stand currently (admittedly subjective in the opinion of the authors)? The attempt of a summary, being aware that this is too broad and entirely domain-specific, is shown in Table 2. It can be seen that we are not quite there yet—but we are constantly moving forward, albeit slowly.

**Table 2:** Assessment of Transcriptomics and Cell Morphology/Cell Painting Data to Make Practical Impact in Drug Discovery Projects

**Endpoint property**	**Gene expression**	**Cell painting**
Reproducibility	Depends on platform; technical reproducibility often sufficient, while biological reproducibility can represent problems	Reproducibility of assay setup across different types of *experimental platforms* can be non-trivial; once set up, reproducibility is generally fit for the purpose (although, based on personal communication, person-to-person variation between technicians can influence results)
Predictivity of signal to *in vivo* endpoints	Needs to be established on a case-by-case basis; some work has been done over the past 10–15 years, but much remains to be done	Translation to *in vivo* endpoints still largely needs to be established (which is a currently intense area of research)
Causality	Data itself is not causal, e.g., time-course analysis needs to be performed to work toward causality	Data itself is not causal, e.g., time-course analysis needs to be performed to work toward causality
Wide applicability/necessity of tailoring	Potentially widely applicable readout, but applicability domain still needs to be established further	Potentially widely applicable readout, but applicability domain still needs to be established further
Standardized data processing pipeline	Data processing somewhat standardized but depends heavily on type of input data available (microarray data processing is more standardized than bulk RNA-Seq processing, which is more standardized than processing of single-cell data currently)	Given that a standard protocol for Cell Painting assays exists, this has in principle fewer degrees of freedom than in the case of gene expression data, but no definite standards exist currently w.r.t. normalization, removing highly correlated features, etc.
Interpretability/allows inclusion of prior information	Gene expression data is inherently interpretable on the gene and pathways level; allows for inclusion of pathway information	Cell morphology data is inherently (by itself) not interpretable on the gene level; does not *per se* allow for the inclusion of pathway information (but readouts can be linked to landmark compounds etc.)
Speed, cost	Depends on platform; newer platforms have low cost and are fully automatable	Generally low running cost but high upfront investment (financially and related to setting up machinery)

Of course, biology does not only exist in the distinct layers of gene expression data and cell morphology data (and beyond)—and also links between domains exist, which have been analyzed before^74,75^ with the aim to understand the relative information content related to particular areas of chemical and response space better. In one of the studies,^75^ it has been found that Cell Painting measures fewer distinct groups of features compared to L1000 gene expression readouts, where hence L1000 transcriptomics data was also able to distinguish a larger number of modes of action. In general, both assays provided complementary information to each other in this work.

In biological assays often concentration/dose, time point, and cell line are key parameters that can significantly change results. For the Cell Painting assay, that by default uses the U2OS osteosarcoma cell line, recent work^76^ has shown relative insensitivity to cell line choice (as opposed to a work cited earlier which studied combination effects of a smaller set of compounds in four cell lines^68^) for 14 phenotypic reference chemicals across six biologically diverse human-derived cell lines (U2OS, MCF7, HepG2, A549, HTB-9, and ARPE-19 cells). Image acquisition settings and cell segmentation parameters needed to be adjusted for each cell type, but not the cytochemistry protocol. The more biological responses generalize across assay parameters such as cell line, the easier the above “Applicability Domain” question becomes—however, there will be natural limits to it, given that different cells are *meant* to respond differently to different stimuli after some thresholds have been surpassed.

For achieving *in vivo* relevant predictions, extrapolation to the whole organism system at therapeutically relevant concentrations is required. To this end, recently *in vivo* PK models have been published to predict *in vivo* PK directly based on chemical structure,^77−79^ but the question of how to integrate (a) the dose and (b) a detected signal in proxy space and to link this to (c) a decision-making point deserves further attention.

To really advance the field, consortia will be needed to explore biological readout space and its utilization to predict *in vivo* relevant endpoints further—some of those consortia have operated in the transcriptomics data in the past, such as the QSTAR Consortium supported by Janssen,^44^ while in the Cell Painting area the JUMP consortium has made data available to the public domain on a large scale. Those consortia will achieve best results if they include aspects of prospective experimental design across the chemical space to be tested, agreement on the biological setup of data generation (cell line, dose, time point, etc.) and data handling, as well as clear links to *in vivo* relevant toxicity endpoints. This is in contrast to consortia based only on “data sharing” of data that has been generated for a completely different purpose in the past, and where chemistry used, assay setup, and annotations employed do not usually match a given new purpose. Likewise, the sharing of results needs to be performed on a large scale, to understand better which type of readout is predictive for which type of *in vivo* endpoint.

**Figure 1:**
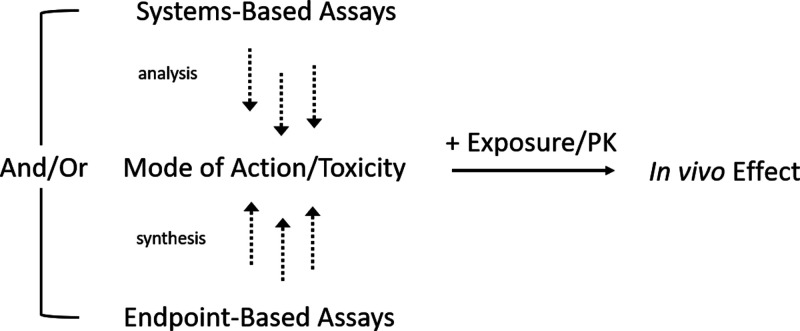
Architecture of *in vivo*-relevant endpoint models, combining either systems-based assay readouts (top), such as transcriptomics or Cell Painting based readouts with PK/exposure models to arrive at *in vivo* relevant predictions, or endpoint-based assays with PK models for this purpose (bottom). The great advantage of using systems-based assays would be that no individual assays are required for each individual toxicity (or also efficacy) endpoint, but that rather a systems-based readout is able to be predictive for a variety of *in vivo* effects in a quantitative manner.

Due to the size of biological hypothesis space, this will in most cases likely necessitate the inclusion of prior information in addition to data, as well as including PK information to assess any type of efficacy- and safety-related endpoints, leading to two possible model architectures described in Figure 1: (1) Either safety endpoints, such as DILI, require a certain amount of mechanistic understanding, leading to the insight that BSEP inhibition is one of the mechanistic factors that can lead to DILI; then relevant chemical space gets tested with respect to this endpoint and models get combined with PK models to make *in vivo* relevant predictions. However, here for every endpoint (or toxicity pathway) experimental data is required. (2) Alternatively, and this is the great promise of *systems-based assays* such as the ones presented here, several or even many modes of toxicity can be captured in a single assay, where the toxicity-relevant signal is combined with PK/exposure information to arrive at predictions relevant for the *in vivo* situation. To understand better which endpoints to be measured independently in an assay (in the first case), or which variables to be considered as predictive (in the second case) for the in vivo situation is the subject of intense ongoing research.
